# Ectopic expression of *GmNF-YA8* in Arabidopsis delays flowering *via* modulating the expression of gibberellic acid biosynthesis- and flowering-related genes and promotes lateral root emergence in low phosphorus conditions

**DOI:** 10.3389/fpls.2022.1033938

**Published:** 2022-10-20

**Authors:** Siyan Ou, Zhihao Xu, Cuishan Mai, Bodi Li, Jinxiang Wang

**Affiliations:** ^1^ State Key Laboratory for Conservation and Utilization of Subtropical Agro-bioresources, South China Agricultural University, Guangzhou, China; ^2^ Root Biology Center & College of Natural Resources and Environment, South China Agricultural University, Guangzhou, China; ^3^ Guangdong Laboratory of Lingnan Modern Agricultural Science and Technology, Guangzhou, China

**Keywords:** *GmNF-YA8*, flowering, phosphorus deficiency, lateral roots, gibberellic acid

## Abstract

NUCLEAR FACTOR Y subunit alpha (NF-YA), together with NF-YB and NF-YC, regulates plant growth and development, as well as plant responses to biotic and abiotic stresses. Although extensive studies have examined the functions of NF-YAs in *Arabidopsis thaliana*, the roles of NF- YAs in *Glycinme max* are poorly understood. In this study, we identified a phosphorus (P) starvation-responsive *NF-YA8* in soybean. The expression of *GmNF-YA8* is induced by low P or low nitrogen in leaves, but not by potassium or iron starvation, respectively. GmNF-YA8 is localized in the nucleus and plasma membrane. Ectopic expression of *GmNF-YA8* inhibits plant growth and delayed flowering in *Arabidopsis*. Exogenous application of gibberellic acid (GA) rescues the delayed flowering phenotype in *Arabidopsis* overexpressing *GmNF-YA8* lines GmNF-YA8OE-05 and GmNF-YA8OE-20. Moreover, quantitative real time PCR (qRT-PCR) verified that overexpression of *GmNF-YA8* downregulates *GA20ox2* and *GA3ox2* expression, but upregulates *GA2ox2* and *GA2ox3* that encode enzymes, which inactive bioactive GAs. Consistent with the late flowering phenotype of *Arabidopsis* trangenic lines that overexpress *GmNF-YA8*, the transcript levels of flowering-promoting genes *AP1*, *CO*, *LFY*, and *SOC1* are reduced. In addition, overexpression of *GmNF-YA8* promotes the emergence of lateral root (LR) primordium from epidermis rather than the initiation of LR in low P, and increases the LR density in low nitrogen. Our results provide insights into the roles of *GmNF-YA8*.

## Introduction

The NUCLEAR FACTOR Y (NF-Y) heterotrimeric complexes, which consist of NUCLEAR FACTOR Y alpha (NF-YA), NUCLEAR FACTOR Y beta (NF-YB), and NUCLEAR FACTOR Y gamma (NF-YC), have been reported to bind the the CCAAT box, a *cis*-element presents in about 25% of eukaryotic gene promoters (Mantovani, 1998; Petroni et al., 2012). In mammals and yeast, each NF-Y subunit is encoded by a single gene, but plant genomes contains multiple genes for each subunit. For example, the *Arabidopsis thaliana* genome contains 10 *NF-YA*, 13 *NF-YB*, and 13 *NF-YC* genes. Similarly, dozens of *NF-YA*, *NF-YB*, and *NF-YC* genes have been identified in rice (*Oryza sativa*), wheat (*Triticum aestivum*), Brachypodium, and *Glycine max* (Stephenson et al., 2007; Thirumurugan et al., 2008; Cao et al., 2011). The soybean genome contains 21 *GmNF-YA*, 32 *GmNF-YB*, and 15 *GmNF-YC* genes (Quach et al., 2015), suggesting that these genes have more diverse roles in soybean than in *Arabidopsis*. However, the functions of the soybean *NF-YA* remain elusive.

Past studies in *Arabidopsis* have revealed that *NF-YA* genes play crucial roles in plant growth and development (Nelson et al., 2007; Li et al., 2008; Petroni et al., 2012). For example, *AtNF-YAs* regulate seed germination. Overexpression of *AtNA-YA1* or *AtNF-YA9* promotes seed germination in the presence of exogenous abscisic acid (ABA) (Siriwardana et al., 2014), but overexpression of *AtNF-YA2*, *AtNF-YA5*, *AtNF-YA8*, or *AtNF-YA10* delays seed germination (Mu et al., 2013; Siriwardana et al., 2014). The *AtNF-YA5* mutant *nf-ya5* does not respond to blue light, and AtNF-YA5 appears to activate *Lhcb1.3* in response to blue light (Warpeha et al., 2007). *AtNF-YA2* is also documented to be involved in photomorphogenesis, and in the dark, transcription of *AtNF-YA2* is repressed and AtNF-YA2 protein is degraded (Myers et al., 2016). *AtNF-YA2* and *AtNF-YA10* are both targets of miR169; overexpressing a miR169-resistant allele of *AtNF-YA2* results in higher density of lateral root (LR), but does not affect primary root (PR) growth (Sorin et al., 2014). Overexpression of *AtNF-YA2*, *AtNF-YA7*, or *AtNF-YA10* results in dwarfing of both seedlings and adult plants, and in smaller siliques and seeds (Leyva-González et al., 2012). Previous studies revealed that some AtNF-YAs are negative regulators of flowering (Wenkel et al., 2006). Overexpression of *AtNF-YA1*, *AtNF-YA4*, *AtNF-YA7*, or *AtNF-YA9* leads to late flowering (Wenkel et al., 2006; Siriwardana et al., 2016). By contrast, AtNF-YA2 seem to be a positive regulator of flowering, and only the heterodimer of AtNF-YA2 and Atnf-YA6, but not AtNF-YA2 or AtNF-YA6, binds the CCAAT-box in promoter of *FT*, thus promoting *FT* expression and early flowering (Siriwardana et al., 2016). Whereas another study reported that AtNF-YA2, AtNF-YB2, and AtNF-YC9 bind to the promoter of *SUPPRESSOR OF OVEREXPRESSION OF CONSTANS1* (*SOC1*) (Hou et al., 2014; Gnesutta et al., 2017), but REPRESSOR OF ga1-3 (RGA), a typical DELLA protein, interacts with AtNF-YA2 and prevents its binding to the promoter of flowering gene *SOC1* (Hou et al., 2014).

A growing body evidence suggests that NF-YAs regulate plants’ responses to abiotic stress. In *Arabidopsis*, overexpressing *AtNF-YA1* lines are hypersensitive to salt stress and ABA during the early post-germination growth stages (Li et al., 2013). *AtNF-YA5* has been reported to be induced by drought, accordingly loss of function of *AtNF-YA5* results in increased water loss in drought conditions (Li et al., 2008). *AtNF-YA10* is downregulated by salinity and ABA, and overexpression of *AtNF-YA10* results in increased sensitivity to salinity and decreased sensitivity to ABA (Ma et al., 2015). In rice, the abundance of *OsNF-YA7* mRNA increases in response to drought, and overexpression of *OsNF-YA7* increases drought tolerance in an ABA-independent manner (Lee et al., 2015). Similarly, transcription of *OsNF-YA8* is induced by drought and its overexpression in rice plants improves their drought tolerance *via* ABA-independent approach. In contrast, *OsNF- YA4* expression is not increased by drought stress, but highly induced by ABA (Lee et al., 2015). The transcript level of *GmNF-YA8* is stimulated by water deficiency (Quach et al., 2015), whereas the role of which in drought tolerance need to be deciphered. In addition, *AtNF-YA5*, *OsNF-YA8*, *GmNF-YA10*, and *GmNF-YA3* are also targeted by miR169 (Li et al., 2008; Zhao et al., 2009; Xu et al., 2013; Ni et al., 2013). These results indicate that transcription of NF-YA is tightly regulated in plants.

*NF-YA*s are involved in plant nutrition. In *Arabidopsis*, transcription of *AtNF-YA2*, *AtNF-YA3*, *AtNF-YA5*, or *AtNF-YA8* is induced by low nitrogen (N) stress in both roots and shoots, and in line with this, the the expression of miR169 is reduced in N-starved roots and shoots (Zhao et al., 2011). Overexpressing *MIR169a* in *Arabidopsis* increases sensitivity to low N stress and decreases the total N contents of roots and leaves (Zhao et al., 2011). Microarray analysis showed that the expression of *AtNF-YA2*, *AtNF-YA3*, *AtNF-YA5*, *AtNF-YA7*, or *AtNF-YA10* is stimulated by low phosphate (Pi) stress (Misson et al., 2005), and further qRT-PCR verified that *AtNF-YA2*, *AtNF-YA7*, And *AtNF-YA10* are induced by Pi deficiency (Leyva-González et al., 2012). Whereas the responses of soybean *NF-YA* family to Pi deficiency remain elusive.

Several studies have been carried out to investigate the functions of *Glycine max NF-YA*s (*GmNF-YA*s). The 21 *GmNF-YA* genes can be divided into three subgroups, and seven of the genes are induced by drought (Quach et al., 2015). *GmNF-YA3* is induced by ABA, NaCl, and cold, and ectopic expression of this gene (renamed as *GmNF-YA12* in 2015 (Quach et al., 2015) in *Arabidopsis* reduces water loss by modulating the expression of *AtABA1*, *AtABA2*, *AtABI1*, and *AtABI2*, thereby increasing tolerance to drought (Ni et al., 2013). More recently, *GmNF-YA5* was reported to be positive regulator during drought stress. Expression of *GmNF-YA5* is induced by drought in an ABA-dependent manner, and the transgenic expression of *GmNF-YA5* enhances the drought tolerance of *Arabidopsis* and soybean (Ma et al., 2020). *GmNF-YA1a* and *1b* have been observed to positively regulate arbuscular mycorrhization (Schaarschmidt et al., 2013). Phosphorus (P) is one of essential macroelements for plant growth and development. Low P stress delays flowering and decreases yield of crops in agriculture (Jiang et al., 2007; Nord and Lynch, 2008). Plants cope with Pi starvation by remodeling of root system architecture with increased number of LRs and LR length, shorter PR, and denser root hair to boost the uptake of Pi from growth medium (López-Bucio et al., 2002; Pérez-Torres et al., 2008). Some classic transcription factors involved in P nutrition such as PHR1 and WRKY6 have been identified in *Arabidopsis*.

In 2013, We identified low Pi-responsive microRNAs and targets of them in soybean at genomic level, and found that soybean miR69 family members target *NF-YA*s (Xu et al., 2013). To further deepen our understanding to soybean’s responses to low P stress and screen low Pi- responsive transcription factors, we initially used RNA-seq experiment to screen long-term (14 days) Pi deficiency-responsive genes in soybean leaves at genomic level. Hundreds of genes induced or repressed by low P in soybean leaves have been identified, including dozens of transcription factor genes. Among them, one of *GmNF-YA*s, namely *GmNF-YA8*, is induced by low P. Given that low P stress delays flowering (Jiang et al., 2007) and overexpression of *NF-YA*s in *Arabidopsis* results in dwarfing and late flowering (Leyva-González et al., 2012), we are curious about the function of *GmNF-YA8* in P nutrition and flowering. In this study, we confirmed that *GmNF-YA8* is induced by low P stress by quantitative real time PCR (qRT-PCR) and established that the gene is also induced by low N stress. GmNF-YA8 is localized in the nucleus and plasma membrane. Ectopic expression of *GmNY-YA8* in *Arabidopsis* delays flowering, and application of exogenous GA rescues the late flowering phenotype. Moreover, overexpression of *GmNF-YA8* decreases *AtGA20ox2* and *AtGA3ox2* expression, and increases *AtGA2ox2* and *AtGA2ox3* expression. *Arabidopsis* plants overexpressing *GmNF-YA8* shows increased LR emergence in low P, and an increased number and density of LRs in low N.

## Materials and methods

### Soybean and arabidopsis growth

Seeds of *Glycine max* genotype YC03-3 were sterilized with 10% NaClO, germinated in sands and transferred when the cotyledon was open and the apical bud had developed. To explore the responses of *GmNF-YA8* to nutrient deficiencies, soybean seedlings were first cultured in complete Hoagland solution (pH 5.9) for 7 days as described (Xu et al., 2013). Then soybean seedlings with the first developed trifoliate leaf were transferred into Pi-replete (HP, 500 µM KH_2_PO_4_), HN (3 mM nitrogen), HK (2 mM potassium), HF (50 µM Fe-EDTA), Pi-deplete (LP, 25 µM KH_2_PO_4_), LN (300 µM nitrogen), LK (250 µM potassium), and LF (0 µM Fe-EDTA) media for 14 days, respectively. Soybean plants were grown in growth chambers with 16h light cycles. Nutrient solutions were aerated 15 min every 3h, and replaced with fresh nutrient solutions every 2 days. As described (Huo et al., 2018), *Arabidopsis* seeds were sterilized with 75% ethanol for 2 minutes followed by 100% ethanol for 2 minutes. Then seeds were sown on half strength MS media containing 1% sucrose and 0.8% agar (pH 5.8). Square petri dishes were maintained in a growth chamber with a 16h light (100 µE m^-2^ s^-1^)/8h dark cycle and with a temperature cycle of 23°C light/21°C darkness. Additionally, stratified *Arabidopsis* seeds were sown in soil and cultured in a growth chamber with a 16h light (100 µE m^-2^ s^-1^)/8h dark cycle and with a temperature cycle of 23° C light/21° C darkness. For the low P and low N treatment, sterilized *Arabidopsis* seeds were sown in half strength MS medium, and stratified in −4° C for 2 days. At 3 days after germination, the seedlings were transferred into different media that contains 500 µM KH_2_PO_4_ (HP), or 0 µM KH_2_PO_4_ (LP), or 10 mM KNO_3_ and 1 mM NH_4_NO_3_ (HN) or 100 µM KNO_3_ and 10 µM NH_4_NO_3_ (LN), respectively. After 7 days, the treated seedlings were scanned to measure the PR length, the average length of first-order LR, and the number of lateral roots and lateral root primordium.

### Quantification of fresh weight, concentrations of soluble phosphate (Pi) and total phosphorus (P) of soybean seedlings.

The sampled soybean plants that were subjected to low P stress for 14 days as described were weighted to quantify the fresh weight, including root and leaves fresh weight. Accordingly, the ratio of root to shoot was determined.

Soybean seedlings were dried at 105°C for 30 min, and then oven-dried at 75°C prior to weighting. The methods described (Zhou et al., 2008) were strictly followed to measure the concentration of soluble Pi and total P.

### Subcellular localization of GmNF-YA8

To explore the subcellular localization of GmNF-YA8, we constructed the fusion protein of GFP with GmNF-YA8. The open reading fragment of *GmNF-YA8* was amplified and then recombined into the pMDC43 vector based on the method as described (Peng et al., 2016). Agrobacterium tumefaciens mediated transient expression in *Nicotiana benthamiana* leaves was conducted as described (Peng et al., 2016). The Agrobacterium GV3101 strain harboring the constructs of GFP-GmNF-YA8 was inoculated in YEP medium and incubated for 24 h at 28°C. After two days, the green fluorescence in the transformed tobacco epidermal cells was detected at 488 nm by confocal laser scanning microscopy (LSM780, Zeiss, Jena, Germany).

### Extraction of genomic DNA, RNA and reserve transcription of mRNA

RNA from Arabidopsis and soybean seedlings was extracted with TRIzol method. RNA was transcribed through MLV-transcriptase. DNA was extracted with CTAB method. Other molecular experiments were based on the standard methods as described (Huo et al., 2018).

### Vector of construction and transformation of *Arabidopsis*

The plasmid for ectopic expressing *GmNF-YA8* was constructed based on standard molecular biology protocols and gateway-based system (Curtis and Grossniklaus, 2003). The open reading frame of *GmNF-YA8* was amplified and cloned into the pMDC32 plasmid. *Arabidopsis* was transformed through floral dipping as described (Clough and Bent, 1998). Single copy T-DNA insertion transgenic lines were screened in growth media containing 50 µg/ml hygromycin. The single copy T-DNA insertion homeozygous T3 generation was used in this study. The RNA was extracted from transgenic lines and the expression levels of *GmNF-YA8* was further assessed by qRT-PCR.

### qRT-PCR analysis

To quantify transcript level of *GmNF-YA8* under different nutrition conditions, qRT-PCR was used. The roots and leaves of soybean cultivar YC03-3 seedlings that were subjected to nutrient stresses were quickly sampled in liquid N, and stored at −80°C. Total RNA extraction and cDNA synthesis were based on standard method as described (Huo et al., 2018). *Glycine max EF1a* (*GmEF1*a) was used to normalize qRT-PCR data (Xu et al., 2013).

The expression levels of flowering- and GA-related genes, as well as four LR emergence-related genes (*AtLAX3*, *AtPIP2;1*, *AtPLA1* and *AtPME1*) were assessed. The rosette leaves of twelve- day-old Col-0 plants and overexpression lines GmNF-YA8OE-05 and GmNF-YA8OE-20 in Col-0 background grown in soils were sampled and total RNA was extracted with Trizol method. cDNAs were obtained with reverse transcriptase-mediated reactions. The transcript abundance of the above-mentioned genes was determined *via* qRT-PCR, and *AtEF1a* was used as a reference gene to normalize qRT-PCR results (Huo et al., 2018). All specific qRT-PCR and clone primer pairs are listed in **Table S1**.

### Quantification of bolting, flowering time, number of rosette leaves, and root-related parameters

The height of the bolt and of *Arabidopsis* plants was measured with a ruler. The flowering time was determined based on the number of days from sowing to the first flower opening. The number of rosette leaves was counted manually. The *Arabidopsis* seedlings in media were scanned, and then the PR length was determined with ImageJ. The *Arabidopsis* LRs were counted and LR primodium (LRP) were measured using a steromicroscopy.

### Data analysis

All data were analyzed with R. Student’s *t*-test was employed to compare differences. R 3.0.1 package (R Core Team, 2018), ggplot2 (Wickham, 2016) and ggpubr (Kassambara, 2019) were used to draw figures, respectively.

## Results

### Low P inhibits soybean seedling growth, and decreases the concentration of solubale and total phosphorus in soybean roots and leaves

**Figure 1:**
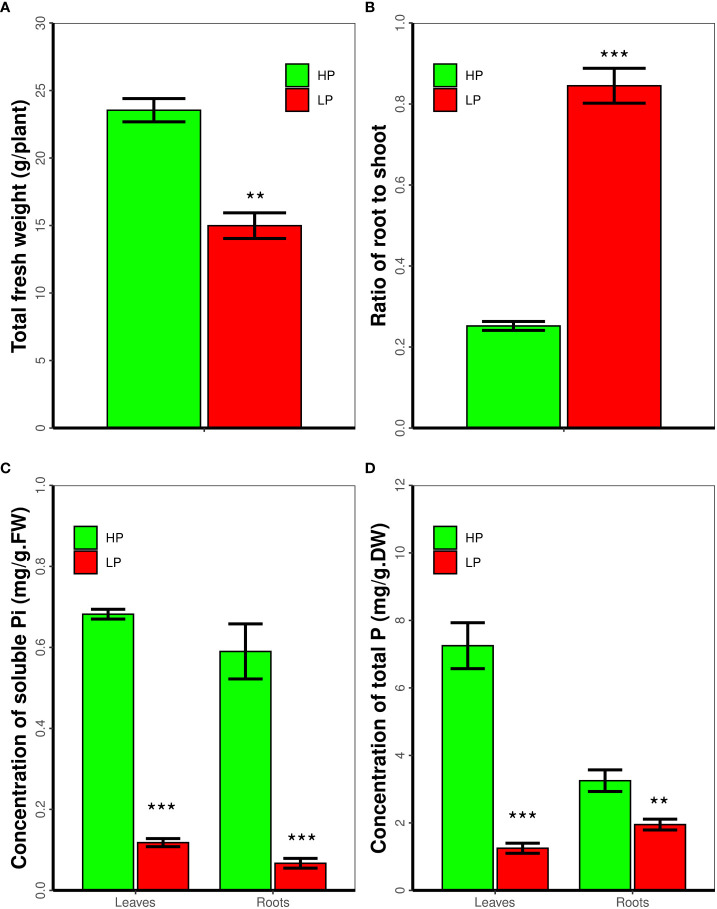
Low P stress inhibits soybean seedling growth and decreases the concentration of soluble Pi and total P. Seven-day-old soybean seedling were subjected to low P stress for 14 day. The Fresh weight of roots and shoots, the ratio of root to shoot, and the concentration of soluble phosphorus and total phosphorus was measured. Results are means ± SE from three independent experiments. Students’*t*-test was used to compare difference. **P < 0.01; ***P < 0.001.

As reported, Pi deficiency inhibit plant growth and development. Accordingly we measure the biomass of soybean seedlings that subjected to low P for 14 days. As shown in **Figure 1A**, in contrast to high P, low P inhibits soybean seedling growth, the total fresh weight of soybean plant in low P is 63.7 percent of that in high P. As expected, low P increases the ratio of root to shoot (**Figure 1B**). Moreover we measured the contents of soluble and total P in soybean plants. Low P treatment significantly decreases the concentration of soluble Pi and total P in soybean roots and leaves, respectively. The concentration of soluble Pi in leaves and roots in high P conditions is 5.8 and 8.8 folds of that in low P, respectively (*P* < 0.01, **Figure 1C**). The concentration of total P in leaves and roots in high P is 5.8 and 1.7 times to that in low P, respectively (*P* < 0.01, **Figure 1D**).

### The expression of *GmNF-YA8* is induced by low P and low N

**Figure 2:**
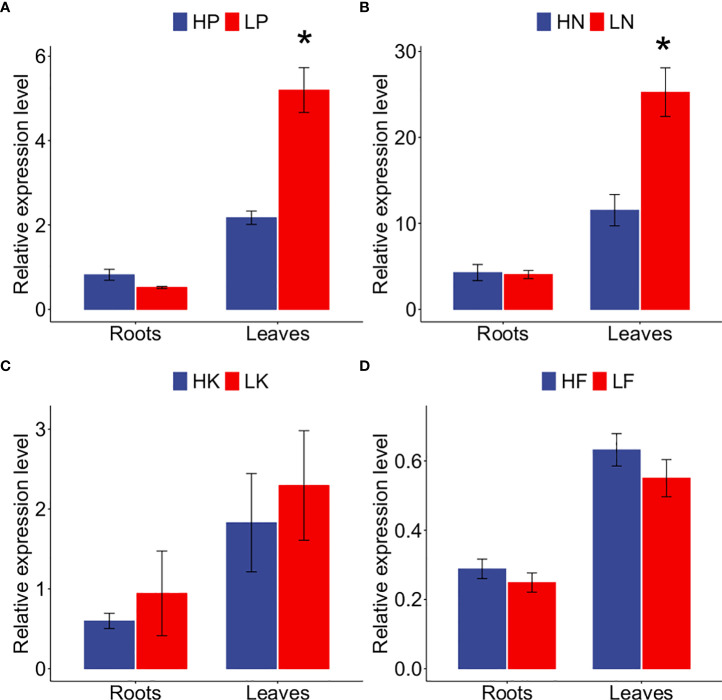
Responses of *GmNF-YA8* to long-term nutrient starvation in soybean roots and leaves. **(A)** phosphorus (P) starvation; **(B)** nitrogen (N) starvation; **(C)** Potassium (K) starvation; **(D)** iron (Fe) starvation. HP, 500 µM KH 2 PO 4; LP, 25 µM KH_2_ PO_4_; HN, 3mM nitrogen; LN, 300 µM nitrogen, HK, 2 mM potassium; LK, 250 µM potassium; HF, 50 µM Fe-EDTA; LF, 0 µM Fe-EDTA. Results are means ± SE from 3 independent experiments. Student’s *t*-test were used to explore the differences between control and nutrient deficiency conditions (*P < 0.05).

As previously reported, *NF-YA* genes are responsive to low P or low N stress in *Arabidopsis* (Zhao et al., 2011; Leyva-González et al., 2012). Although *GmNF-YA8 (Glyma.09g023800)* is induced by drought (Quach et al., 2015), its responses to nutrient deficiencies were not known. Based on our soybean leaf RNA-seq results (deposited in NCBI, accession number: PRJNA489734), we established that *GmNF-YA8* was induced by long-term Pi starvation. Given the responsiveness of *GmNF-YA8* to Pi deficiency, we want to know whether the gene responds to other nutrient deficiencies, specifically N, potassium (K), and iron (Fe). We used qRT-PCR to measure *GmNF- YA8* transcript levels in leaves and roots of soybean plants that had been subjected to long-term (14 days) nutrient deficiencies. Pi starvation (LP) caused a 2.4-fold increase in *GmNF-YA8* transcript levels Relative to the Pi-replete (HP) control in leaves (*P* < 0.05) but not in roots (**Figure 2A**). Under low N (LN), the transcript level of *GmNF-YA8* in leaves were induced 1.19 folds relative to high N (HN) controls (*P* < 0.05, **Figure 2B**). By contrast, low K (LK) and low Fe (LF) did not induce *GmNF-YA8* expression in either leaves or roots (**Figure 2C, D**).

**Figure 3:**
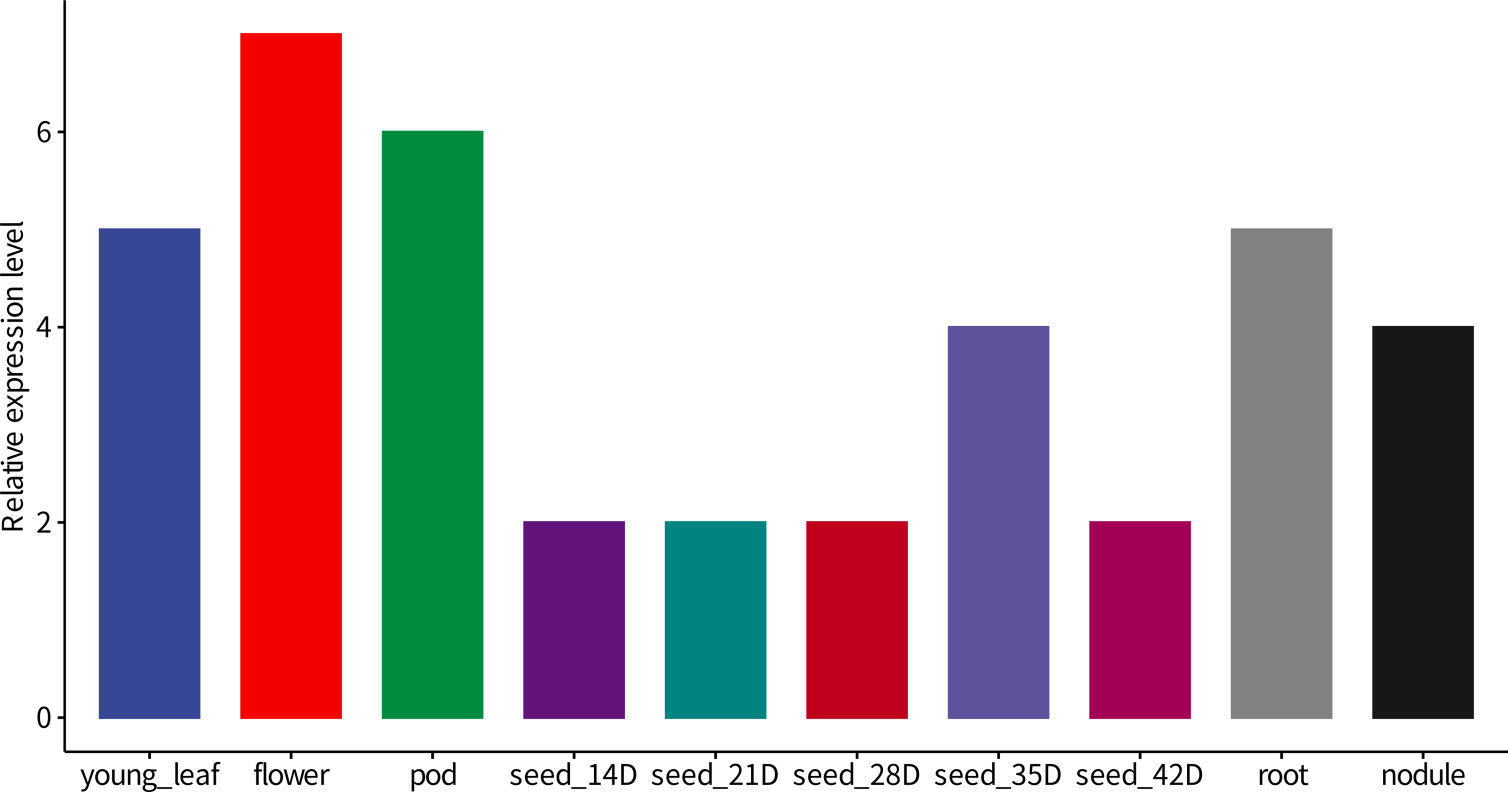
The relative transcript level of *GmNF-YA8* in different soybean organs Base on public RNA-seq data (www.soybase.org), the relative transcript level Reads Per Kilobase Per Million mapped reads (RPKM) of *GmNF-YA8* was shown. POD, one-cm long pod; D, Day after fertilization.

We are also interested in the transcript level of *GmNF-YA8* in soybean organs. Based on the public data (www.soybase.org), the transcripts of *GmNF-YA8* in flower is the highest, followed by that in one-cm pod, young leaf, and root. the expression of *GmNA-YA8* is also detected in seeds and nodule (**Figure 3**). This suggests that *GmNF-YA8* is constitutively expressed in soybean organ and possibly play important role in growth and development. In addition, based on the public RNA-seq data (http://ipf.sustech.edu.cn/pub/soybean/), we found that *GmNA-YA8* is induced by low P, and respond to dehydration, and day length (**Supplementary Sheet 1**). These results imply that *GmNF-YA8* plays versatile roles in response to environmental factors.

### GmNF-YA8 is localized to the nucleus and cell membrane

**Figure 4:**
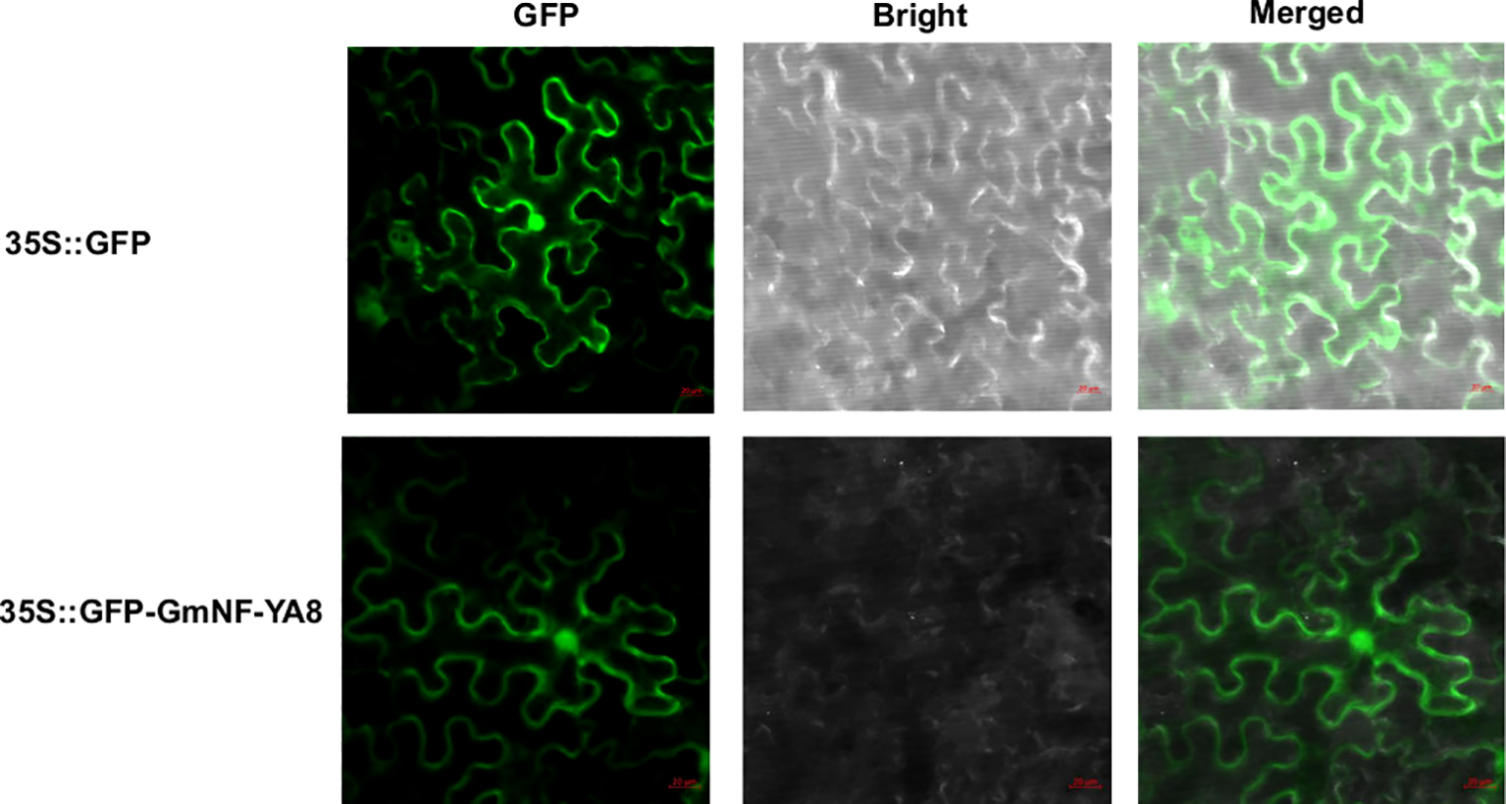
GmNF-YA8 is localized in the nucleus and plasma membrane. GFP signal was detected in transformed *Nicotiana benthamiana* leaf epidermis cells at 488 nm by laser confocal miscroscopy. GFP: GFP channel; Bright: light channel; Merged: the overlap of GFP channel and light channel. 35S::GFP: pMDC43 empty vector containing GFP; 35S::GFP-GmNF-YA8: pMDC43- GmNF-YA8 vector containing the fusion of GFP with GmNF-YA8.

Bioinformatics analysis indicates that *GmNF-YA8* encodes a protein with 219 amino acids (www.phytozome.org), which is predicated by PSORT (www.psort.org) to be localized in the nucleus. Given that *NF-YAs* act as transcription factor in plant cell (Li et al., 2008), we are curious about the subcellular localization of *GmNF-YA8*. To this end, we constructed the fusion protein of GFP-GmNF-YA8 using the gateway vector pMDC43. We then transformed *Nicotiana benthamiana* leaf epidermal cells with *Agrobacterium tumefaciens* GV3101 that contains the construct encoding GFP-GmNF-YA8. After two days, we detected the green fluorescence signals in the nucleus and plasma membrane by laser confocal microscopy (**Figure 4**). This suggests that *GmNF-YA8* is localized in the nucleus and plasma membrane.

### Ectopic expression of *GmNF-YA8* in *Arabidopsis* inhibits stem growth and delays bolting and flowering

To determine the function of *GmNF-YA8*, we overexpressed *GmNF-YA8*, driven by the constitutive 35S promoter in *Arabidopsis* Col-0 wild-type plants. By screening for hygromycin resistance, we identified dozens of T1 transgenic lines. To identify single T-DNA insertion transgenic *Arabidopsis* lines, the T2 seeds from individual T1 plants were sown in half strength MS medium with 25 µg/mL hygromycin and screened after 10 days based on the segregation ratio (3:1) of hygromycin resistance gene. Accordingly, homozygous transgenic lines were also determined in the T3 generation based on hygromycin resistance phenotype. We thus selected two single-copy T- DNA insertion homozygous lines, namely GmNF-YA8-05 and GmNF-YA8-20, and used qRT-PCR to verify that they were transgenic lines (**Figure S1**). Thus these two lines were used for subsequent experiments.

**Figure 5:**
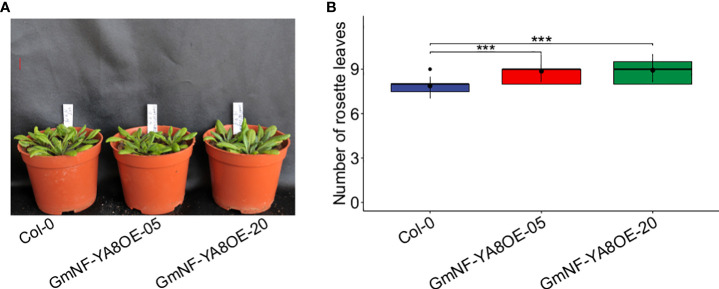
Ectopic expression of *GmNF-YA8* delays bolting in *Arabidopsis* **(A)** Photos were taken when the bolt in 20-day-old Col-0 plants was one-cm high, the transgenic lines had not yet bolted. GmNF-YA8OE-05 and GmNF-YA8OE-20 indicates the transgenic lines that overexpress *GmNF- YA8*, respectively. Scale bar, 1 cm. **(B)** The number of rosettes was counted in each 12 Col-0, GmNF-YA8OE- **(B)** 05, and GmNF-YA8OE-20 plants respectively, when the bolt was one-cm high. Student’s *t*-test was used to compare the difference between Col-0, GmNF-YA8OE-05 and GmNF-YA8OE-20 plants (* *P*< 0.05; ***P < 0.001).

Under long-day (16h light) conditions, bolting was delayed in the transgenic expressing lines relative to non-transformed Col-0 plants (**Figure 5A**). When the stems of Col-0 plants were one-cm high, no obvious bolts were seen in GmNF-YA8OE-05 and GmNF-YA8OE-20 plants. Moreover, Col-0 plants had fewer rosette leaves at the time of bolting than did GmNF-YA8OE-05 and GmNF-YA8OE-20 plants when they bolted (**Figure 5B**).

**Figure 6:**
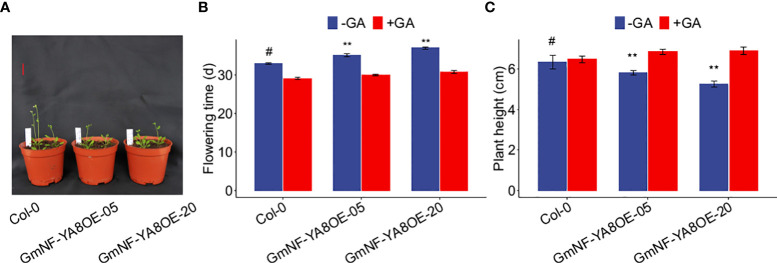
Ectopic expression *GmNF-YA8* delays flowering in *Arabidopsis* **(A)** Photographs were taken when the first flower had opened in Col-0, but not in GmNF-YA8OE-05 and GmNF-YA8OE-20 plants. Scale bar, 2 cm. **(B)** The flowering time was measured when the first flower was opened in Col-0, GmNF-YA8OE-05 and GmNF-YA8OE-20, respectively. **(C)** Plant height was measured when the first flower was opened. Results are means ± SE from each 12 Col-0, GmNF-YA8OE-05 and GmNF-YA8OE-20 plants, respectively. Difference between Col-0 and the transgenic lines GmNF-YA8OE-05 and GmNF-YA8OE-20 were compared using Student’s *t*-test (**P < 0.01; #, the control).

As the bolting of overexpressing *GmNF-YA8* lines was delayed (**Figure 5A**), we deduced that flowering time of overexpressing *GmNF-YA8* lines is also delayed. To verify this, we further explored the effect of ectopic expression of *GmNF-YA8* on flowering time, defined as the number of days when the first flower is opening as described by previous studies (Wenkel et al., 2006; Siriwardana et al., 2016). In control conditions (no GA treatment), GmNF-YA8OE-05 and GmNF-YA8OE-20 plants flowered later than Col-0 plants (*P* < 0.05; **Figure 6A, B**). Application of 100 µM GA_3_ promoted flowering in Col-0, GmNF-YA8OE-05, and GmNF-YA8OE-20 plants, and abolished the difference between the control and transgenic plants (**Figure 6B**). The plant height was also measured when the first flower of the plants had open. In contrast to Col-0 seedlings, the plant height of GmNF-YA8OE-05 and GmNF-YA8OE-20 seedlings was shorter in control conditions (no GA treatment), application of GA obviously increased plant height of GmNF-YA8OE-05 and GmNF-YA8OE-20 plants and masked the difference between Col-0, GmNF- YA8OE-05 and GmNF-YA8OE-20 plants (**Figure 6C**). Collectively, these results indicate that ectopic expression of *GmNF-YA8* delays flowering by decreasing endogenous GA levels.

**Figure 7:**
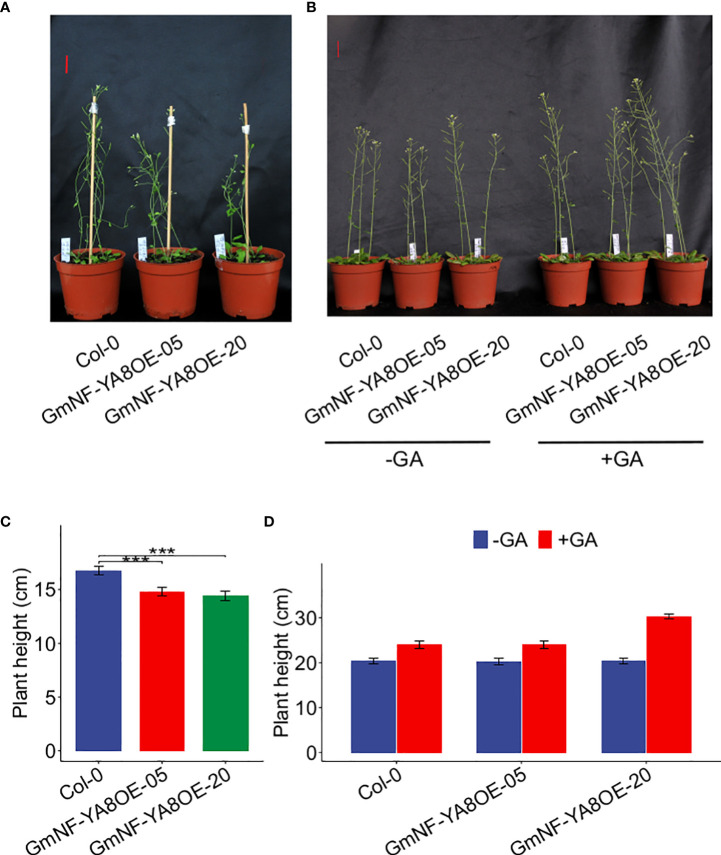
Ectopic expression of *GmNF-YA8* decreases *Arabidopsis* plant height The plant height of the non-transformed Col-0 control and transgenic lines GmNF-YA8OE-05 and GmNF- YA8OE-20 was measured on the 25th day after germination (DAG) **(A, B)** and 35 DAG **(C, D)**, respectively. Results are means ± SE from each 12 Col-0, GmNF-YA8OE-05, and GmNF-YA8OE-20 plants, respectively. Student’s *t*-test was used to compare the differences between Col-0 and the transgenic lines GmNF-YA8OE-05 and GmNF-YA8OE-20 (***P < 0.001). Scale bar, 2 cm.

We also measured plant height at 25 and 35 days after germination (DAG). Under normal conditions (no GA treatment), GmNF-YA8OE-05 and GmNF-YA8OE-20 plants were shorter than Col-0 plants at 25 DAG (**Figures 7A, B**). The height of GmNF-YA8OE-05 and GmNF-YA8OE-20 plants was 88.49% and 86.21% s of Col-0 plants, respectively (**Figure 7B**). Interestingly, there was no significant difference between the height of Col-0, GmNF-YA8OE-05, and GmNF-YA8OE-20 plants at 35 DAG. Application of exogenous 100 µM GA_3_ increased the stem growth of Col-0, GmNF-YA8OE-05, and GmNF-YA8OE-20 plants, more obviously in GmNF-YA8OE-20 plants (**Figures 7C, D**). These results indicate that ectopic expression of *GmNF-YA8* in *Arabidopsis* inhibits stem growth in young plants but does not affect GA responsiveness.

### Ectopic expression *GmNF-YA8* affects the transcription of GA biosynthesis- and deactivation-related genes

**Figure 8:**
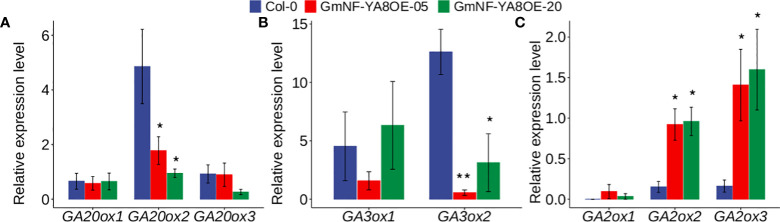
Effects of overexpressing *GmNF-YA8* in Arabidopsis on the transcript levels of GA biosynthesis- and deactivation-related genes. **(A)**: *GA20ox* gene family; **(B)**: *GA3ox* gene family; **(C)**:*GA2ox* gene family. Results are means ± SE from three independent experiments. Student’s *t*-test was used to compare the difference between Col-0, and transgenic lines GmNF-YA8OE-05 and GmNF-YA8OE-20 (*P < 0.05; **P < 0.01).

As well documented, GAs play important roles in stem growth and flowering and delaying leaf senescence (Hedden and Thomas, 2012; Colebrook et al., 2014). Here we established that ectopic expression of *GmNF-YA8* delayed flowering of *Arabidopsis* (**Figures 6A, B**), and that application of GA rescued flowering (**Figure 6B**). We thus deduced that ectopic expression of *GmNF-YA8* affects GA biosynthesis and/or deactivation. To reveal the underlying molecular mechanisms, we measured the expression levels of GA biosynthesis- and deactivation-related genes in the rosette leaves of 12-day-old *Arabidopsis* seedlings. As reported, *GA20ox* and *GA3ox* are involved in the biosynthesis of active GAs, whereas *GA2ox* catalyzes the conversion of bioactive GA1 and GA4 to inactive GAs such as GA8 and GA34 (Ogawa et al., 2003; Hedden and Thomas, 2012). We found that transcript levels of *GA20ox2*, but not *GA20ox1* and *GA20ox3* were significantly lower in GmNF- YA8OE-5 and GmNF-YA8OE-20 plants than that in Col-0 plants (**Figure 8A**). In addition, the transcript abundance of *GA3ox2*, but not *GA3ox1*, was much lower in GmNF-YA8OE-5 and GmNF- YA8OE-20 plants than that in Col-0 plants (**Figure 8B**). As for *GA2ox* expression, ectopic expression of *GmNF-YA8* increased the transcript levels of *GA2ox2* and *GA2ox3*, but not *GA2ox1* (**Figure 8C**). These data suggest that ectopic expression of *GmNF-YA8* decreases the production of active GAs and promotes the conversion of active GAs to inactive GAs.

### Ectopic expression of *GmNF-YA8* affects the transcription of flowering-related genes

**Figure 9:**
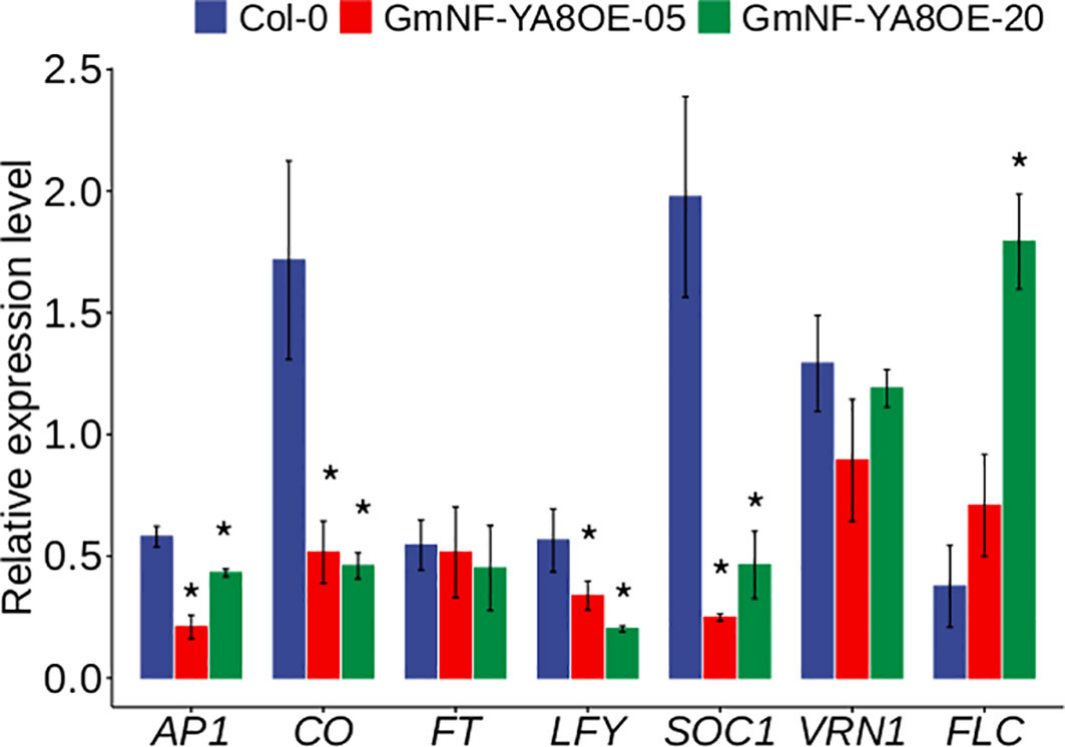
Effects of ectopic expression of *GmNF-YA8* on the expression levels of flowering-related genes in *Arabidopsis*. Results are means ± SE from three independent experiments. Student’s *t*- tests was used to compare the differences between Col-0 and the transgenic lines GmNF-YA8OE- 05 and GmNF-YA8OE-20 (*P < 0.05).

Previous studies have shown that *NF-YAs* positively or negatively regulate flowering in *Arabidopsis* (Siriwardana et al., 2016). As revealed, APETALA1 (AP1), CONSTANS (CO), FLOWERING LOCUS T (FT), LEAFY (LFY), SUPPRESSOR OF OVEREXPRESSION OF CONSTANS1 (SOC1), and VRN (Chandler et al., 1996; Blazquez et al., 1998; Ng and Yanofsky, 2001; Levy et al., 2002; Moon et al., 2003) promote flowering, whereas FLOWERING LOCUS C (FLC) inhibits flowering in *Arabidopsis* (Michaels and Amasino, 1999; Bastow et al., 2004). Given that ectopic expression of soybean *NF-YA8* delayed flowering in *Arabidopsis* (**Figure 4**), we wondered whether ectopic expression of *GmNF-YA8* represses flowering by modulating the expression of flowering-related genes. As shown, the transcripts of *AP1*, *CO*, *LFY*, and *SOC1* were significantly downregulated in GmNF-YA8OE-05 and GmNF-YA8OE-20 relative to Col-0, whereas *FLC* was significantly upregulated in GmNF-YA8OE-20 line relative to Col-0 (**Figure 9**). These results indicate that *GmNF-YA8* delays flowering probably by decreasing the transcription of *AP1*, *CO*, *LFY*, and *SOC1*.

### Ectopic expression of *GmNF-YA8* promotes lateral root emergence under low P

**Figure 10:**
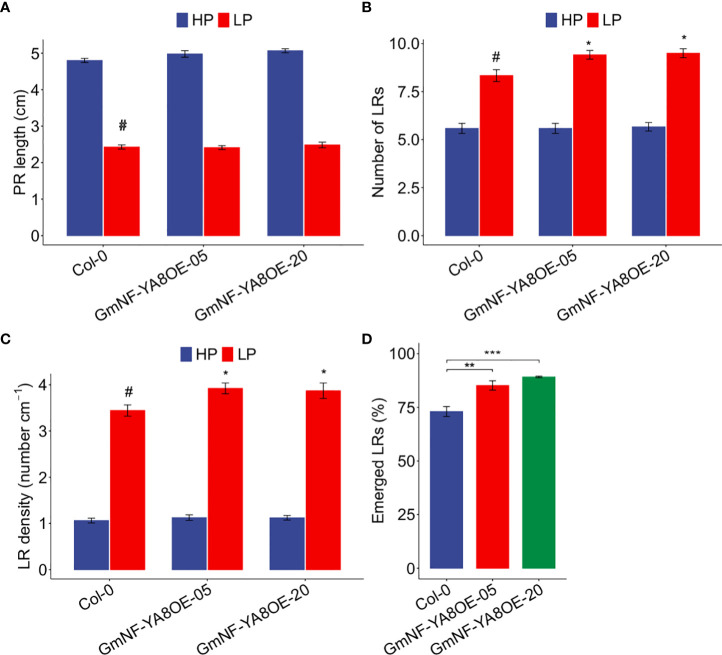
Ectopic expression of *GmNF-YA8* increases number of lateral roots (LRs) under low P (LP) conditions, and boosted the percentage of emerged LRs under LP. **(A)**Primary root (PR) length; **(B)** Number of LRs; **(C)** LR density; **(D)** Emerged LRs (%). Results are means ± SE of three independent experiments. Student’s *t*-test was used to compare the differences between Col-0 and the transgenic lines GmNF-YA8OE-05 and GmNF-YA8OE-20 (*P < 0.05; **P < 0.01; ***P < 0.001; #, the control).

In soybean, *GmNF-YA8* expression was induced by low P and low N (**Figure 2A, B**). We thus investigated whether ectopic expression of *GmNF-YA8* affects low P responses in *Arabidopsis*, in particular the remodeling of root system architecture (López-Bucio et al., 2002). To reveal the potential roles of *GmNF-YA8* in root growth and development under low P conditions, we grew the overexpressing *GmNF-YA8* lines in high P and low P and measured the PR length, the number of LRs and LR density. Consistent with previous studies (Jiang et al., 2007), low P clearly inhibited PR growth in Col-0, GmNF-YA8OE-05, and GmNF-YA8OE-20 plants, respectively (**Figure 10A**). In low P, the number of LR was significantly increased in the transgenic lines by 13.42% in GmNF- YA8OE-05 and 14.44% in GmNF-YA8OE-20 relative to Col-0 (*P* < 0.05, **Figure 10B**), but in high P there was no difference between Col-0 and transgenic lines. Additionally, the LR density in GmNF- YA8OE-05 and GmNF-YA8OE-20 was higher than in Col-0 (*P* < 0.05, **Figure 10C**). There are three possibilities to explain overexpressing *GmNF-YA8* results in more emerged LRs: by increasing initiation of LR primordium (LRP), by stimulating the penetration of LRP from epidermis, or by both mechanisms. To distinguish these possibilities, we counted the total number of LRP and the number of emerged LR. No difference was observed in the total number of LRP between Col-0 and the transgenic lines, but the percentage of emerged LRs was 85% in GmNF-YA8OE-05 (*P* < 0.01) and 89% in GmNF-YA8OE-20 (*P* < 0.001), in contrast to 73% in Col-0 (**Figure 10D**). These results suggest that *GmNF-YA8* does not promote initiation of LRP but does promote the emergence of LRs from the epidermis in low P conditions.

Lateral root emergence from epidermis is influenced by LIKE AUXIN-UPTAKE CARRIERS3 (LAX3), PECTIN METHYL ESTERASE1 (PME1, PECTIN LYASES1 (PLA1, and PLASMA MEMBERANE INTRINSIC PROTEIN2;1 (PIP2;1) (Laskowski et al., 2006; Swarup et al., 2008; Péret et al., 2012; Vilches-Barro and Maizel, 2015) in *Arabidopsis*. As revealed, *AtPME1* catalyzes the degradation of pectin (Lavenus et al., 2013). *PLA1* transcript levels are very high when LRP penetrate from epidermis (Laskowski et al., 2006). As an auxin influx carrier, LAX3 is responsible for the accumulation of auxin in cortex and epidermal cells and upregulates the expression of *XTR6* that is involved in LR growth and development (Swarup et al., 2008; Péret et al., 2012). By contrast, transcription of *AtPIP2;1* is repressed when LRP emerged from epidermis (Péret et al., 2012; Lavenus et al., 2013). To gain a better understanding to how *GmNF-YA8* influences LR emergence, we examined the expression of these four genes in PR of wild type Col-0 and overexpressing *GmNF-YA8* plants that were cultured in high P and low P for 7 days. Because no LRP were observed in the first 0.4 cm of the primary root tip, we discarded this region of the PR and collected the remainder for extraction of total RNA. There was no difference in the transcript abundance of *AtPME1*, *AtPLA1*, *AtLAX3*, and *AtPIP2;1* between the transgenic lines GmNF- YA8OE-05, GmNF-YA8OE-20 and Col-0 (**Figure S2**). Considering that these four genes are tightly regulated at the temporal and spatial levels, future experiments will need to measure their transcript levels in epidermis, cortex, and endodermis.

### Ectopic expression of *GmNF-YA8* increases lateral root number, length, and density under low N

**Figure 11:**
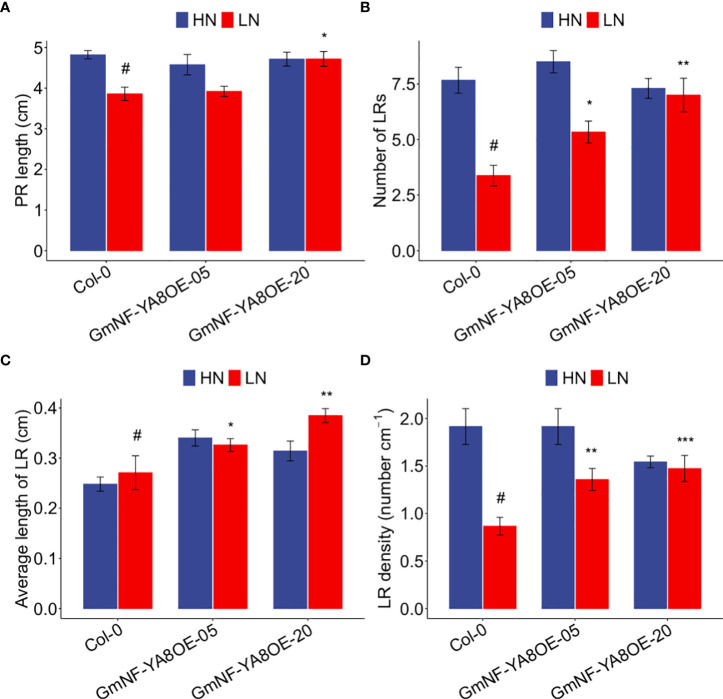
Ectopic expression of *GmNF-YA8* increases number of lateral roots (LRs), average length of first-order LRs, and LR density in low N. **(A)** PR length; **(B)** LR number; **(C)** average length of first-order LRs; **(D)** LR density. Results are means ± SE from three independent experiments. Student’s *t*-test was used to assess the differences between Col-0 and the transgenic lines GmNF-YA8OE-05 and GmNF-YA8OE-20 (*P < 0.05; **P < 0.01; ***P < 0.001; #, the control).

NF-YAs and miR169 have been reported to be involved in low N responses (Zhao et al., 2011). Because *GmNF-YA8* was induced by low N (**Figure 1B**), we thus explored the role of this gene in low N responses. We grew Col-0, GmNF-YA8OE-05, and GmNF-YA8OE-20 plants in high N and low N, and then measured their root-related parameters. In high N, we observed no difference between Col-0 and the ectopic expression lines in PR length (**Figure 11A**), number of LRs (**Figure 11B**), average length of the first-order (primary) LRs in each seedling (**Figure 11C**), and LR density (**Figure 11D**). In low N, by contrast, the number of LRs was increased by 58.02% in GmNF-YA8OE05 (*P* < 0.05) and by 107.40% in GmNF-YA8OE-20 plants (*P* < 0.01, **Figure 11B**) relative to Col-0; the average length of first-order LRs in each seedling was boosted by 20.35% in GmNF-YA8OE-05 (*P* < 0.05) and by 42.04% in GmNF-YA8OE-20 plants (*P* < 0.01, **Figure 11C**); and LR density was increased by 56.77% in GmNF-YA8OE-05 (*P* < 0.01) and by 70.15% in GmNF-YA8OE-20 seedlings (*P* < 0.001, **Figure 11D**). These results suggest that *GmNF-YA8* play active roles in remodeling of root system in response to N deficiency.

## Discussion

It is well documented that NF-YA interacts with NF-YB and NF-YC to form heterotrimeric transcription factors that regulate the expression of genes involved in plant growth, development, and stress tolerance (Siriwardana et al., 2016). The *Glycine max* genome encodes 21 NF-YAs, 32 NF-YBs, and 15 NF-YCs (Quach et al., 2015), with the potential to form a huge number of different heterotrimeric combinations. Past studies demonstrated that expression of *GmNF-YA3* is induced by drought in soybean and that ectopic expression of *GmNF-YA3* in *Arabidopsis* increases drought tolerance (Ni et al., 2013). In addition, overexpressing *GmNF-YA5* enhances tolerance of *Arabidopsis* and soybean to drought, respectively (Ma et al., 2020). In this study, we revealed that low P stress increased the ratio of root to shoot (**Figure 1B**), and decreased the concentration of soluble Pi and total P in soybean roots or leaves (**Figure 1C, D**). *GmNF-YA8* is induced by low P and low N stress in soybean leaves (**Figure 2**), and GmNF-YA8 is localized in the nucleus and plasma membrane (**Figure 4**). We further showed that overexpression of *GmNF-YA8* in *Arabidopsis* delays bolting (**Figure 5**), flowering (**Figure 6**), and stem growth (**Figure 7**) *via* modulating the expression of GA biosynthesis- and flowering-related genes (**Figure 8**-**9**). Ectopic expression of *GmNF-YA8* in *Arabidopsis* promoted LR emergence in low P (**Figure 10**),as well as boosting LR growth and density under low N conditions (**Figure 11**). To our knowledge,this is the first report on the role of soybean *NF-YA* in flowering, and root growth and development in low P.

NF-YAs are key players in plant nutrition. Microarray analysis indicates that *AtNF-YA2*, *AtNF-YA3*, *AtNF-YA5*, *AtNF-YA7*, and *AtNF-YA10* are induced by low P availability. An increase in *AtNF-YA2*, *AtNF-YA8*, and *AtNF-YA10* transcript levels was detected by qRT-PCR 4 days after germination in low P, low N, and high sucrose conditions. *AtNF-YA3* and *AtNF-YA5* showed a similar response to high sucrose but no change in response to low N and low P conditions (Leyva-González et al., 2012). Our initial RNA-seq results revealed that *GmNF-YA8* was induced in soybean leaves by P starvation (deposited with NCBI, accession number: PRJNA489734). In addition, our RNA-seq analysis showed that *Glyma.15G129900*, another NF-YA gene, is also induced by low P in soybean leaves. In the present study, qRT-PCR confirmed that *GmNF-YA8* was induced in leaves but not roots in low P (**Figure 2A**). These results suggest that *GmNF-YA8* modulates systemic responses of soybean to Pi deficiency. Of course, the identification of low Pi- responsive *NF-YA*s in soybean roots is of importance to understand the role of *GmNF-YA* in local P signaling. In *Arabidopsis*, the expression of *AtNF-YA2*, *AtNF-YA3*, *AtNF-YA5*, and *AtNF-YA8* is induced by N starvation (Wang et al., 2003; Zhao et al., 2011). Consistently, we revealed that *GmNF-YA8* is induced by lone-term low N stress (**Figure 1B**). Of note, the 2-kb-long promoter regions of *AtNRT1.1* and *AtNRT2.1* contain CCAAT *cis*-elements (Zhao et al., 2011), suggesting that these genes are regulated by *NF-YAs* in *Arabidopsis*. Up to now, it is not clear whether *GmNF-YA8* regulates the transcription of nitrate transporter in soybean. Our findings imply that the involvement of *NF-YAs* in N and P signaling is conserved across plant species. Considering the responses of *GmNF-YA8* to P and N starvation, identifying genes that are regulated by *GmNF-YA8* at the transcription level is crucial to understand the roles of *GmNF-YA8* in P and N nutrition, especially those GA biosynthesis- and flowering-related genes in soybean. As reported, plant transcription factor NF-YAs bind CCAAT motif to regulate gene transcription. Give that overexpressing *GmNF-YA8* alters the expression level of GA- and flowering-related genes in Arabidopsis, we thus analyzed the CCAAT *cis*-element in the promoter region of related *Arabidopsis* genes. As expected, one to nine CCAAT (or ATTGG) motifs were found in tested genes (**Table S2**). For instance, one CCAAT motif is found in promoter of *AtGA2ox1*, nine CCAAT motifs in promoter of *AtGA3ox1* (**Table S2**). Thus we deduced that overexressing *GmNF-YA8* delays flowering *via* modulating the activities of GA-and flowering-related genes at transcription level. Moreove it is also necessary to screen transcription factors that directly bind the *cis*-element in the promoter of *GmNF-YA8.*

NF-YAs have been report to be nuclear protein. For instance, AtNF-YA5, GmNF-YA3 and ZmNF-YA3 are localized in nucleus (Li et al., 2008; Ni et al., 2013; Su et al., 2018). Consistently, here we found that GmNF-YA8 is localized in nucleus by tobacco leaf transient transformation experiment (**Figure 4**), indicating GmNF-YA8 seem to act as transcription factor. On the other hand, the GFP-GmNF-YA8 fusion protein can also be detected in the cell membrane (**Figure 4**). We noticed that some transcription factors are localized to plasma membrane of plant cells (Liu et al., 2014; Yang et al., 2020).

As reported, overexpression of *AtNF-YA2*, *AtNF-YA8*, or *AtNF-YA10* in *Arabidopsis* results in a dwarf phenotype (Leyva-González et al., 2012). In line with this, we observed that ectopic expression of *GmNF-YA8* in *Arabidopsis* delayed bolting (**Figure 5**) and decreased seedling height, especially during the early stages of growth (**Figure 6**, **Figure 7**). The application of GA_3_ rescues the slow stem growth (**Figure 6**, **Figure 7**). Moreover, we detected a decrease in the transcript levels of GA biosynthesis-related genes such as *GA20ox2* and *GA3ox2*, and an increase in those of *GA2ox2* and *GA2ox3* that are involved in GA inactivation (**Figure 8**). Thus, *Arabidopsis* plants overexpressing *GmNF-YA8* might have reduced GA biosynthesis and increased GA metabolism. Moreover, genes encoding xyloglucan endotransglucosylase-hydrolases (XTHs) and expansins (EXPs), which are involved in cell elongation, have been observed to be strongly downregulated in overexpressing *AtNF-YA2*, *AtNF-YA7*, or *AtNF-YA10* Arabidopsis lines (Leyva-González et al., 2012). Hence, it remains to be determined whether the levels of *XTH* and *EXP* transcripts are also reduced in Arabidopsis plants overexpressing *GmNF-YA8*.

Previous studies revealed that *NF-YA* positively or negatively regulate flowering (Siriwardana et al., 2016). Here we revealed that ectopic expression of *GmNF-YA8* in Arabidopsis delays flowering (**Figure 6B**). Consistently, overexpressing *AtNF-YA7* or *NF-YA9* also delays flowering in Arabidopsis (Siriwardana et al., 2016). However, overexpressing *AtNF-YA2* or *NF-YA6* results in early flowering (Siriwardana et al., 2016). Interestingly, ZmNF-YA3 forms complex with CO-like and FPF1, and thus binds the promoter of FT like 12 to promote flowering in maize (Su et al., 2018). AtNF-YA2 and AtNF-YA6 heterotrimerize with NF-YB2 and NF-YC3 *in vitro* to bind the -5.3 kb CCAAT box of FLOWERING LOCUS T, thus promoting flowering (Siriwardana et al., 2016). Interestingly, the transcription of flowering-related genes such as *AP1*, *CO*, *LFY*, and *SOC1* was reduced by the overexpression of *GmNF-YA8* (**Figure 9**). Hence, it is possible that GmNF-YA8 together with NF-YB and NF-YC, even other NF-YA, regulates flowering *via* its binding to the *cis*- element of GA biosynthesis- and flowering-related genes in *Arabidopsis*. Further ChIP-seq experiments will be used to verify this.

As reported, low P stress results in the reduction of bioactive GA *(GA4) via* upregulating *GA20ox1* and *GA2ox2*, and application of exogenous GA promotes *Arabidopsis* flowering under low P conditions (Jiang et al., 2007). Moreover, suboptimal P availability delays bolting, flowering and maturation in different *Arabidopsis* genotypes grown in soils (Nord and Lynch, 2008). Here, we revealed that long-term low P stress induced *GmNF-YA8* expression in soybean leaves but not roots (**Figure 1**), and overexpressing *GmNF-YA8* delays flowering in *Arabidopsis* (**Figure 6**). Hence we hypothesize that *GmNF-YA8* is involved in the flowering process of soybean when subjected to low P stress. Low P stress promotes LR emergence and LR density (López-Bucio et al., 2002; Pérez-Torres et al., 2008). We observed that overexpressing *GmNF-YA8* increased the density of LRs when compared to Col-0 plants in low P (**Figure 10**). However, no difference was detected in the total number of LRP between Col-0 and transgenic lines (**Figure 10**). Ectopic expression of *GmNF-YA8* only stimulated the emergence of LRP but not the initiation of LRP (**Figure 10**). We also explored the expression of *AtPME1*, *AtPLA1*, *AtLAX3*, and *AtPIP2* in low P, which are involved in LR emergence. To our surprise, the transcript abundance of these genes were not significantly affected by overexpressing *GmNF-YA8* (**Figure S2**). *AtPME1*, *AtPLA1*, *AtPLA2*, and *AtEXP1* are induced by auxin when LRPs are emerging or have emerged from the cortex and epidermis cells (Laskowski et al., 2006; Swarup et al., 2008), whereas *AtPIP2;1* is downregulated by auxin in cortex cells during LR emergence stage (Péret et al., 2012). Thus, LR emergence-related genes are tightly regulated in a spatial-and temporal-dependent manner. Here, we just detected the expression levels of *AtPLA1*, *AtPME1*, *AtLAX3*, and *AtPIP2;1* in the rooting zone (**Figure S2**). Hence, we can not rule out the possibility that these genes are expressed at different levels in the epidermis, cortex, and endodermis cells between Col-0 and GmNF-YA8OE-05 and GmNF-YA8OE-20 line. Of note, no difference was detected between Col-0 and the overexpressing *GmNF-YA8* lines (GmNF-YA8OE-05 and GmNF-YA8OE-20) in high N (10 mM KNO_3_ plus 1 mm NH_4_NO_3_) with respect to PR length, average length of first-order (or primary) LRs, number of LRs, and LR density. Whereas the number of LRs, average length of first-order LRs, LR density were boosted in transgenic lines under low N conditions (0.1 mM KNO_3_ plus 0.01 mm NH_4_NO_3_) (**Figure 11**). This suggests that *GmNF-YA8* might improve the tolerance of transgenic *Arabidopsis* to low N stress *via* regulating LR emergence and growth. Different results have been reported based on different experimental systems; for instance, LR elongation and PR growth are decreased by high N (1 mM nitrate), but LR density does not appear to be affected by nitrate availability (0.01-1 mM nitrate) (Linkohr et al., 2002). High N (10 mM KNO_3_ plus 5 mM glutamine) decreases the number of LRs, LR length and density in *Arabidopsis* Col-0, but low N (0.5 mM KNO_3_ plus 5 mM glutamine) lightly increases the number and density of LRP and LRs (Tranbarger et al., 2003).

In Arabidopsis, *AtNF-YA2* and *AtNF-YA10* modulates LR development. Both *AtNF-YA2* and *AtNF-YA10* are highly expressed in the pericycle, followed in vasculature of elongation zone of PR. Overexpression of miR169-resistant *AtNF-YA2* leads to the increase in emerged LRs and total LRPs (Sorin et al., 2014). In Lotus, *LjNF-YA1* and *LjNF-YB1* have been observed to regulate LR development. Overexpression of *LjNF-YA1* results in abnormal LR tips, and extra cell division is taken place in the pericycle that is the origin of LRP (Siefers et al., 2009). Although we did not detect the expression domains of *GmNF-YA8* in PR and LR at tissue level, overexpression of *GmNF-YA8* has no role in PR and LR growth and development in normal nutrient conditions (**Figure 10**-**11**). Given that *Glycine max* genome has 21 *NF-YAs*, it is necessary to screen other *NF-YAs* that regulate soybean root growth and development *via* detailed transcriptome analysis together with gene editing.

Taken together, we have characterized the functions of *GmNF-YA8* gene *via* ectopic expression. Ectopic overexpressing *GmNF-YA8* in *Arabidopsis* delayed bolting, flowering, and repressing stem growth *via* affecting the expression of GA-biosynthesis-related genes (*GA20ox2*, *GA20ox3*, *GA3ox2*, *GA20ox2*, *GA20ox3*) and flowering-related genes (*AP1*, *CO*, *LFY*, and *SOC1*), and promoted LR emergence in low P and low N. Hence, *GmNF-YA8* appears to have potential applications in breeding soybean with improved N and P effiiciency in agriculture.

## Data availability statement

The datasets presented in this study can be found in online repositories. The names of the repository/repositories and accession number(s) can be found in the article/**Supplementary Material**.

## Author contributions

SO, ZX participated in the study design, carried out the experiments and data analysis. CM, BL carried out the experiments and analyzed data. JW conceived, designed experiments, analyzed data, and authored the manuscript. All authors have contributed to the article and approved the submitted manuscript.

## Funding

This study was partially supported by National Key Research and Development Program of China (No.2021YFF1000500) and Natural Science Foundation of Guangdong Province, China (No. 2017A030313102).

## Acknowledgments

We thank Jennifer Mach for her comments and help in English writing.

## Conflict of interest

The authors declare that the research was conducted in the absence of any commercial or financial relationships that could be construed as a potential conflict of interest.

## Publisher’s note

All claims expressed in this article are solely those of the authors and do not necessarily represent those of their affiliated organizations, or those of the publisher, the editors and the reviewers. Any product that may be evaluated in this article, or claim that may be made by its manufacturer, is not guaranteed or endorsed by the publisher.

## Supplementary material

Supplementary Figure 1 Identification of over-expressing *GmNG-YA8* in Arabidopsis wild type Col-0 background. The #5 (GmNF-YA8OE-05) and #20 (GmNF-YA8OE-20) indicate the two single copy T-DNA insertion lines. Total RNA was extracted from Col-0 and transgenic Arabidopsis lines, respectively. The quantitative real-time PCR (qRT-PCR) was employed to explore the transcript level of GmNF-YA8 in Arabidopsis. Arabidopsis *EF1a* was used as house-keeping gene to normalize PCR results.

Supplementary Figure 2 Transcript levels of *AtLAX3*, *AtPME1*, *AtPIP2*, and *AtPLA1* in rooting zone of PR.

Supplementary Table 1 List of primer pairs used in this study.

Supplementary Table 2 List of CCAAT motifs in the promoter region of GA- and flowering-related genes.

Supplementary Sheet 1 Fold change of *GmNF-YA8* to different treatments. RNA-seq data is from public soybean RNA-seq database (http://ipf.sustech.edu.cn/pub/soybean/), which includes 4,085 *Glycine max* RNA-Seq libraries.
