# Supplementary material for: Ectopic expression of GmNF-YA8 in Arabidopsis delays flowering via modulating the expression of gibberellic acid biosynthesis- and flowering-related genes and promotes lateral root emergence in low phosphorus conditions
Source: Front Plant Sci. 2022 Oct 20;13:1033938. doi: 10.3389/fpls.2022.1033938 (PMC9630906; doi:10.3389/fpls.2022.1033938)
Supplement: Supplementary Figure 1 — Identification of over-expressing GmNG-YA8 in Arabidopsis wild type Col-0 background. The #5 (GmNF-YA8OE-05) and #20 (GmNF-YA8OE-20) indicate the two single copy T-DNA insertion lines. Total RNA was extracted from Col-0 and transgenic Arabidopsis lines, respectively. The quantitative real-time PCR (qRT-PCR) was employed to explore the transcript level of GmNF-YA8 in Arabidopsis. Arabidopsis EF1a was used as house-keeping gene to normalize PCR results. [file DataSheet_2.pdf]

SUPPLEMENTARY FIGURE 1

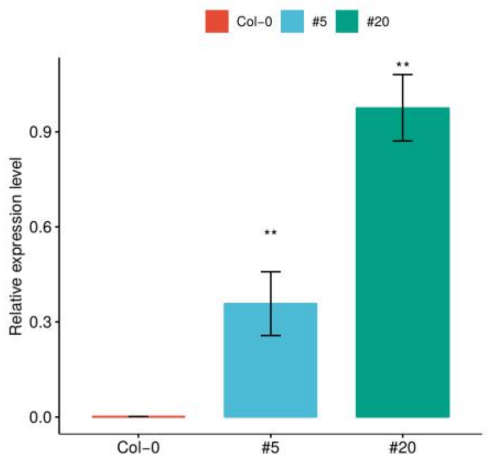

Identification of over-expressing *GmNG-YA8* in Arabidopsis wild type Col-0 background. The #5 (GmNF-YA8OE-05) and #20 (GmNF-YA8OE-20) indicate the two single copy T-DNA insertion lines. Total RNA was extracted from Col-0 and transgenic Arabidopsis lines, respectively. The quantitative real-time PCR (qRT-PCR) was employed to explore the transcript level of GmNF-YA8 in Arabidopsis. Arabidopsis *EF-1a* was used as house-keeping gene to normalize PCR results. Student's *t*-test was used to compare the difference (** $P <$ 0.01)
