# Supplementary figures and images for: Ectopic expression of GmNF-YA8 in Arabidopsis delays flowering via modulating the expression of gibberellic acid biosynthesis- and flowering-related genes and promotes lateral root emergence in low phosphorus conditions
Source: Front Plant Sci. 2022 Oct 20;13:1033938. doi: 10.3389/fpls.2022.1033938 (PMC9630906; doi:10.3389/fpls.2022.1033938)

## SUPPLEMENTARY FIGURE 2

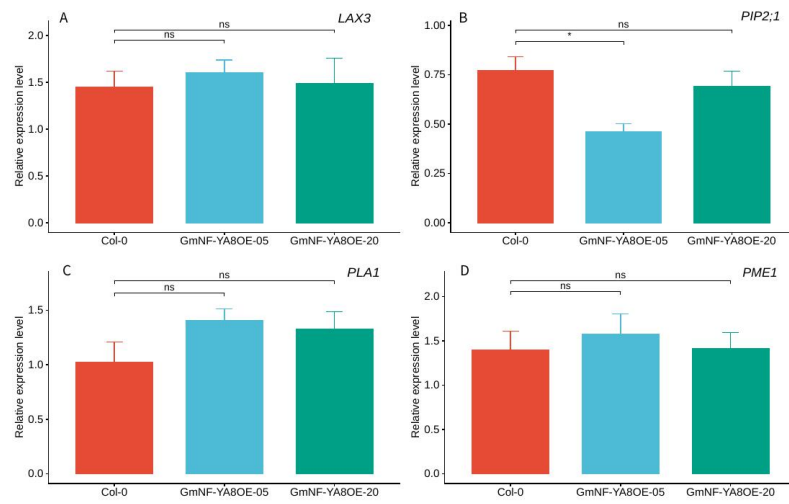

Transcript levels of *AtLAX3*, *AtPME1*, *AtPIP2*, and *AtPLA1* in rooting zone of PR

Supplement: Supplementary Figure 2 — Transcript levels of AtLAX3, AtPME1, AtPIP2, and AtPLA1 in rooting zone of PR. [file DataSheet_3.pdf]
