# Supplementary material for: Ectopic expression of GmNF-YA8 in Arabidopsis delays flowering via modulating the expression of gibberellic acid biosynthesis- and flowering-related genes and promotes lateral root emergence in low phosphorus conditions
Source: Front Plant Sci. 2022 Oct 20;13:1033938. doi: 10.3389/fpls.2022.1033938 (PMC9630906; doi:10.3389/fpls.2022.1033938)
Supplement: Supplementary Table 1 — List of primer pairs used in this study. [file Table_1.pdf]

SUPPLEMENTARY TABLE 1

List of primer pairs used in this study

Primer name	Sequence (5’-3’)	Usage
GmNF-YA8.qF	CATGACCTCTCAGATAATGAAGC	qRT-PCR
GmNF-YA8.qR	TGCAAGTATGGCTTCCGAT	qRT-PCR
OX-GmNF-YA8.oxF	ggggacaagtttgtacaaaaaagcaggcttcTCATGTGTGTAGATTGGGACACTG	Over-expression
OX-GmNF-YA8.oxR	ggggaccactttgtacaagaaagctgggtcCTTAGGATGTTCTATCTGATGGTGC	Overe-xpression
AP1.qF	ATGAGAGGTACTCTTACGCCGA	qRT-PCR
AP1.qR	CAAGTCTTCCCCAAGATAATGC	qRT-PCR
CO.qF	CACTACAACGACAATGGTTCC	qRT-PCR
CO.qR	GGTCAGGTTGTTGCTCTACTG	qRT-PCR
FLC.qF	CTCTACAGCTTCTCCTCCGGCG	qRT-PCR
FLC.qR	GTAGCTCATAGTGTGAACCATAGTTCAGAG	qRT-PCR
FT.qF	CCCTGCTACAACTGGAACAAC	qRT-PCR
FT.qR	CACCCTGGTGCATACACTG	qRT-PCR
LFY.qF	TGAACATCGCTTGTCGTCAT	qRT-PCR
LFY.qR	CGACGATCCGGTACAGCTA	qRT-PCR

SOC1.qF	GTGATCTCCACTCAACAAAAA	qRT-PCR
SOC1.qR	CAACAAGAGAGAAGCAGCTTTA	qRT-PCR
VRN1.qF	GTTACTCCATTCGCATTGGTTATC	qRT-PCR
VRN1.qR	TGTGAGCGGAATCCATGAGAC	qRT-PCR
AtGA2ox1(At1g78440).qF	CGGGAACTTTCAGAAACGC	qRT-PCR
AtGA2ox1(At1g78440).qR	ACATTCTTACCACCATTGG	qRT-PCR
AtGA2ox2(At1g30040).qF	TTTCCGTGAGTCGGTGG	qRT-PCR
AtGA2ox2(At1g30040).qR	CTCCGCCTCTTCCTCCG	qRT-PCR
AtGA2ox3(At2g34500).qF	GCAATTTTCAGAGAGGCAG	qRT-PCR
AtGA2ox3(At2g34500).qR	CTCTTCCTTGACCGGAG	qRT-PCR
AtGA3ox1(At1g15550).qF	CCACGGCGTGCCTTTGG	qRT-PCR
AtGA3ox1(At1g15550).qR	GATATCGCAGTAGTTGAGG	qRT-PCR
AtGA3ox2(At1g80340).qF	CCTCGCGACTTCTCGAC	qRT-PCR
AtGA3ox2(At1g80340).qR	AATAATTTCACAGTATTTGAGG	qRT-PCR
AtGA20ox1(At4g25420).qF	CTCATGAATACACGAGCC	qRT-PCR
AtGA20ox1(At4g25420).qR	TGATACACCTTCCCAAATG	qRT-PCR

AtGA20ox2(At5g51810).qF	ATGCTCACCGTTTGATGG	qRT-PCR
AtGA20ox2(At5g51810).qR	CCTTCCCAAACTGCTCG	qRT-PCR
AtGA20ox3(At5g07200).qF	CCTATCTGCATATGGACTC	qRT-PCR
AtGA20ox3(At5g07200).qR	AAACCTTCCCGAAATCTTC	qRT-PCR
AtLAX3.qF	TCACCATTGCTTCACTCCTTC	qRT-PCR
AtLAX3.qR	AAGCACCATTGTGGTTGGAC	qRT-PCR
AtPIP2;1.qF	TGTGTTTTCCACTTGCTCTTTTG	qRT-PCR
AtPIP2;1.qR	CACAACGCATAAGAACCTCTTTGA	qRT-PCR
AtPLA1.qF	CTTTTAGCAATGATGTGTTTACTC	qRT-PCR
AtPLA1.qR	CCGCTACTTCATCTGGATTC	qRT-PCR
AtPME1.qF	ACGGCGACGTTTGCTATACAAGG	qRT-PCR
AtPME1.qR	TGCTCCCGCGGTGTTTATGATTC	qRT-PCR
GmEF1a.qF	CTGGAGGTTTTGAGGCTGGTAT	qRT-PCR
GmEF1a.qR	CCAAGGGTGAAAGCAAGAAGA	qRT-PCR
AtEF1a.qF	CTGGAGGTTTTGAGGCTGGTAT	qRT-PCR
AtEF1a.qR	CCAAGGGTGAAAGCAAGAAGA	qRT-PCR
