# Supplementary material for: Ectopic expression of GmNF-YA8 in Arabidopsis delays flowering via modulating the expression of gibberellic acid biosynthesis- and flowering-related genes and promotes lateral root emergence in low phosphorus conditions
Source: Front Plant Sci. 2022 Oct 20;13:1033938. doi: 10.3389/fpls.2022.1033938 (PMC9630906; doi:10.3389/fpls.2022.1033938)
Supplement: Supplementary Table 2 — List of CCAAT motifs in the promoter region of GA- and flowering-related genes. [file Table_2.pdf]

SUPPLEMENTARY TABLE 2

List of CCAAT motif in the promoter region of GA- and flowering-related genes

Promoter of Gene	Number of CCAAT（或 ATTGG） motifs	location
AtGA2ox1(At1g78440)	1	-63 ～-59
AtGA2ox2(At1g30040)	4	-1721～-1717,-935～-931,-878～-874,-393～-389
AtGA2ox3(At2g34500)	7	-1904～-1900,-1502～-1498,-1437～-1433,-1279～-1275,-970～-966,-909～-905,-176～-172
AtGA3ox1(At1g15550)	9	-1592～-1588,-1444～-1440,-1170～-1166,-843～-839,-572～-568,-211～-207,-191～-187,-183～-179,-97～-93
AtGA3ox2(At1g80340)	2	-782～-778,-178～-174
AtGA20ox1(At4g25420)	4	-1775～-1771,-1317～-1313,-1310～-1306,-55～-51
AtGA20ox2(At5g51810)	6	-1965～-1961,-1845～-1841,-1336～-1332,-1032～-1028,-797～-793,-563～-559
FLC: (AT5G10140)	8	-1714～-1710,-1657～-1653,-1625～-1621,-1263～-1259,-1245～-1241,-1145～-1141,-1138～-1134,-795～-791
AP1: AT1G69120	5	-1144～-1140,-693～-689,-288～-284,-115～-111,-62～-58
CO: AT5G15840	4	-2316～-2312,-1845～-1841,-1592～-1588,-156～-152
FT: AT1G65480	4	-1615～-1614,-1246～-1237,-1055～-1051,-741～-737

LFY: AT5G61850	5	-1655～-1651,-1612～-1608,-1531～-1527,-888～-884,-757～-753
SOC1:AT2G45660	4	-1580～-1576,-1212～-1208,-1919～-1915,-469～-465
VRN1:AT3G18990	5	-1862～-1858,-1258～-1254,-1067～-1063,-1037～-1033,-988～-984
